# An Overview of Food and Drug Administration Medical Device Legislation and Interplay with Current Medical Practices

**DOI:** 10.7759/cureus.4627

**Published:** 2019-05-09

**Authors:** Abraham Schlauderaff, Kaleigh C Boyer

**Affiliations:** 1 Neurosurgery, Penn State Milton S. Hershey Medical Center, Harrisburg, USA; 2 Miscellaneous, Widener University, Harrisburg, USA

**Keywords:** fda, medical device, medical device legislation

## Abstract

Since the emergence of medical devices, legislation has been developed to allow the Federal Drug Administration (FDA) to oversee their development, marketing, and usage. This paper discusses the history of the FDA’s involvement in medical devices, current approval processes, and several case examples. Additionally, it discusses both short- and long-term effects with unexpected consequences to U.S. health care delivery.

## Introduction and background

Brief overview of historical legislation

The precursor to the Food and Drug Administration (FDA) first emerged in 1906, when President Theodore Roosevelt signed the Pure Food and Drugs Act, which prohibited interstate commerce of misbranded and adulterated food and drugs. In 1938, the Federal Food, Drug, and Cosmetic Act (“FD&C Act”) authorized the FDA to regulate medical products. This allowed the FDA to perform factory inspections and prohibited misbranded marketing of cosmetic and therapeutic medical devices. In 1944, the Public Health Service Act focused on the expansion of biologics and more formal evaluation of laboratories. In 1968, the Radiation Control for Health and Safety Act focused on regulating devices which utilized radiation or magnetic fields. In 1970, President Nixon recruited the director of the National Heart and Lung Institute, Dr. Theodore Cooper, to chair the Cooper Committee to evaluate the need for medical device legislation. The Cooper Committee recommended new specific legislation for devices in a risk-stratified manner [1].

Medical device amendments to the FD&C Act

Based on the Cooper Committee’s recommendations, the first formal legislation was developed in 1976 to strictly oversee medical devices. Medical Device Amendments to the FD&C Act was an intensive classification process that included multiple elements. It sought to assure the safety and effectiveness of medical devices. It risk-stratified medical devices into three classes which were based on inherent device risks: Class I; Class II; and Class III. It required devices developed after May 28, 1976, to go through the Premarket Approval (PMA) and 510(k) premarket notification regulatory pathways. It included the Investigational Device Exemption so that new investigational devices could be studied. Additionally, it developed post-market surveillance once a device had entered the market. Device manufacturers were required to sustain good manufacturing practices and report adverse events to the FDA. If there were concerns with post-market surveillance, the FDA was given the authority to ban a device from the market [1].

Class I Devices

Class I devices are deemed low-risk and are unlikely to cause bodily harm if a malfunction occurs [2]. They are regulated by general (i.e., assurance of safety and effectiveness) controls which are deemed sufficient to maintain safety to the public [3]. Rigorous data-driven FDA approval is not needed for Class I devices, however, if post-marketing surveillance shows a device to be unsafe the FDA is able to remove it from the market. Common Class I devices include tongue depressors, crutches, and blood pressure cuffs [2].

Class II Devices

Class II devices are deemed moderate-risk devices and are unlikely to lead to preposterous bodily harm if a malfunction occurs [2]. They are not only regulated by general controls but additionally special (i.e., requirements for special labeling, performance marketing, and post-market surveillance) controls [3]. Most devices which fall into this category obtain FDA clearance by the premarket notification 510(k) process prior to the device entering the market. The 510(k) premarket notification requires a device to compare itself to a “substantially equivalent” device that has previously been on the market [2]. The 510(k) notice must be submitted 90 days prior to the device entering the market [4]. It does not require the predicate device to be presently on the market, as such, the substantially equivalent device may have been removed from the market due to safety concerns or poor outcomes. Very few 510(k) applications require submission of clinical data as much of the data is oftentimes from rudimentary animal or in vitro experiments. Huerta et al. discussed that less than 1% of 510(k) applications submit clinical data to support substantial equivalence claims [5]. If upon review the FDA deems it necessary they may require more clinical data although this is relatively uncommon. The 510(k) process has recently been controversial and attracted much media attention. It has critically been reviewed where some have deemed it flawed and unreliable [3]. Examples of Class II devices include cardiac monitors, tampons, surgical drapes, and foreign body retrievers [2].

Class III Devices

Class III devices are deemed high-risk devices and may cause serious injury but are intended to significantly modify patient health [2]. General and regulatory controls are not rigorous enough to establish safety and effectiveness so PMA is required to be filed in accordance with section 515 of the FD&C Act prior to marketing a device [2]. The PMA is more rigorous than the 510(k) process, therefore, device manufactures attempt to bypass it if able [3]. The FDA critically appraises the clinical data submitted in the PMA to determine if it is safe and effective and should be placed on the market. The FDA has up to 180 days to review PMA applications. Examples of Class III devices include pacemakers, defibrillators, mechanical heart valves, and endovascular stents [2].

Safe Medical Devices Act

In 1990, the Safe Medical Devices Act (SMDA) legislation was placed to modify the 1976 Medical Device Amendments to the FD&C Act. Prior to this, the post-market surveillance was deemed insufficient so modifications were made. The facilities that utilized devices were required to report adverse events. The FDA was also given the ability to mandate post-market surveillance on permanently implanted devices that could cause serious bodily harm or death. FD&C Act violations could be punished by civil penalties and device withdrawal from the market. It further delineated the definition of “substantially equivalent” which was utilized by the 510(k) pathway. Devices that were developed for rare diseases were more readily able to enter the market with the addition of the Humanitarian Use Device (HUD) and Humanitarian Device Exemption (HDE) programs [1].

Food and Drug Administration Modernization Act

In 1997, the Food and Drug Administration Modernization Act tried to streamline the process by creating the “least burdensome” provisions for premarket review and allowed third parties to perform premarket reviews. New iterations of devices were able to submit clinical data from earlier iterations for premarket submissions. This established the De Novo program which allowed new low-to-moderate risk devices to be classified as Class I or II risks [1].

Medical Device User Fee and Modernization Act

In 2002, the Medical Device User Fee and Modernization Act allowed the FDA to receive fees for medical device premarket submissions and also allowed certain small business to have reduced fees through the small business determination program. It used fees as a way to increase efficiency on evaluated devices [1].

Food and Drug Administration Amendments Act

In 2007, the Food and Drug Administration Amendments Act increased the efficiency of the premarket review process, moved the process to an electronic format, and required each device to bear a unique identification number [1].

Food and Drug Administration Safety and Innovation Act

In 2012, the Food and Drug Administration Safety and Innovation Act allowed collaboration between the United States and foreign governments regulations. It further simplified new low-to-moderate risk devices to be classified as Class I or II and bypass the 510(k) process. It further streamlined and sped up the review times in addition to making the decisions more transparent to device submitters [1].

21st Century Cures Act

In 2016, the 21st Century Cures Act made it easier for new devices to be approved. It included expansion of the FDA’s expedited review policy, increased “least burdensome” reviews, made it easier for devices to receive 510(k) exemption, increased the population required for HUD designation from 4,000 to 8,000, and altered internal review board centralization [1].

## Review

Current Food and Drug Administration device approval process

All new medical devices intended for the public must go through the application process overseen by the FDA’s Center for Devices and Radiological Health (CDRH), which regulates pre- and post-market surveillance of devices. The specific process undertaken by the manufacturer depends on deemed inherent device risk and intended population size. The process undertaken for Class II and III devices was previously discussed, however, there are exceptions for small target populations.

Approval for small target populations

An HUD obtains FDA approval via a separate pathway, under the HDE. HUDs are unique and novel Class III devices that are targeted to treat disease processes with low incidences. In 2016, the 21st Century Cures Act liberalized the requirement from 4,000 to 8,000 target individuals [4]. Unlike PMA, the device manufacturer is not required to submit clinical data due to the difficulty in performing clinical trials with such a limited target patient population. However, they must prove that it is unlikely to pose a great risk to patients and that there is likely significant clinical benefit when utilized. Once approval is obtained as an HDE, it differs from a PMA approval as it requires a device to be classified as an investigational device and its use is limited to the original indications that were submitted to the FDA. Clinicians that place these devices must obtain approval from their local internal review board prior to placement. An example of an approved HUD via the HDE process is the Berlin Heart EXCOR Pediatric Ventricular Assist Device. Ventricular Assist Devices are commonly utilized in the adult population to act as a temporizing measure while transplant-list patients await their turns for placement. This is far less common in the pediatric population which allowed the device’s approval as an HUD [2].

Post-market surveillance

In addition to approving medical devices to be marketed, the FDA is also tasked with monitoring devices that have already been placed on the market. Originally in 1984, the FDA required manufacturers to report adverse events to the FDA. In 1990, the SMDA legislation additionally required clinicians/facilities utilizing devices to report adverse events to both the FDA and the manufacturer. The Medical Device Reporting database is easily searchable and shows all reported adverse events associated with a particular device on the market. In addition to this mandated self-reporting, the FDA may require more formal post-market surveillance programs which the device manufacturer must initiate and report to the FDA within 30 days of market entrance [2]. Many are concerned by this process as it is prone to reporter bias - with an estimated less than 5% complication rate being reported to the FDA [6].

Selected case studies

Biologic Hernia Meshes

Huerta et al. discussed the current issues involved with biologic hernia meshes used by general surgeons. Abdominal hernia repairs are one of the most commonly performed surgeries in the US with approximately 190,000 operations being performed in 2012 [5]. Recurrent abdominal hernias can be a costly complication which led to the development and increased utilization of mesh implants after they were shown to reduce hernia recurrence. Mesh implants, however, are associated with complications, such as bowel erosion or increased infection rates, which led to the development of biological meshes in the 1990s even though there is no high-level data supporting their usage [5]. This shift has become a very profitable industry for device manufacturers with annual revenue estimated at $400 billion [5]. Biologic meshes are Class II devices, and as such, they utilize the 510(k) pathway for market entrance. Due to the ease of comparing a new type of mesh to another “substantially equivalent” mesh, there has been an explosion of costly biologic meshes on the market with questionable clinical benefit [7].

Transvaginal Mesh Devices for Pelvic Organ Prolapse

Henneghan analyzes non-absorbable polypropylene mesh devices which are commonly utilized for the treatment of pelvic organ prolapse and stress incontinence. Originally, these meshes were Class II devices and were substantially equivalent to the Mersilene mesh that was originally developed in the 1950s. Most 510(k) filings have no clinical data or outcomes to support their devices benefits and safety profile. In 2011, reports of vaginal erosions, infections, bladder injury, increased reoperation rates, and organ perforations were associated with these mesh devices. Many manufacturers removed their devices from the market when the FDA required them to submit post-market surveillance data in 2012. After more concerns surfaced, these mesh devices were changed from Class II to Class III devices in 2016. To this day, very limited data exists to substantiate any benefit from using these mesh devices [8].

Metal on Metal Hip Implants

Total hip replacements are one of the most common surgeries performed in the US with approximately 300,000 surgeries performed in 2010 [9]. The popularity of Cobalt-alloy metal on metal total hip arthroplasty devices -particularly in younger patients - stems from their reported longevity. This has led to a regained interested in these devices, and popularization in the 1990s, despite robust clinical data after entering the market via the 510(k) pathway as Class II devices. Cobalt-containing hip implants have recently been quite controversial and have attracted substantial media attention. They have been associated with Cobalt toxicity which includes central nervous system impairment [9], heart disease, and thyroid dysfunction [10]. There have been multiple case series that have reported removal of these devices due to elevated serum Cobalt levels in patients with psychological alterations confirmed with formal testing [9]. Additionally, clinical data has revealed increased revision rates when compared to other implants [3, 8].

Positive and Negative Effects of FDA Legislation

The aforementioned legislation has led to widespread changes in health care. Positive benefits have included a robust expansion of available medical technology and increased treatment options. New areas of medicine have been developed around medical devices such as functional neurosurgery, endovascular neurosurgery, interventional radiology, and interventional cardiology, to name a few. Previous disease processes which were difficult to treat are not associated with as much morbidity or mortality that plagued previous generations. Modifications and improvements to existing devices are able to be promptly performed via the 510(k) process. Development of life-saving devices for small target populations, such as pediatric ventricular assist devices, is continually being performed with improvement in quality of life.

This legislation, however, has received much criticism. Many concerns have emerged for medical device manufacturers due to the lack of oversight and unexpected loopholes. The lack of clinical data required to obtain 510(k) clearance, mass usage of predicate devices that have been removed from the market, and poor post-market surveillance have all surfaced as possible concerns. Widespread use of new expensive devices with questionable benefits may be contributing to soaring U.S. health care costs. The emergence of new medical devices lacking profound benefits dominates discussions surrounding current and future legislation to modify the current process.

## Conclusions

Since the emergence of medical devices in health care, legislation has been developed to allow the FDA to oversee the development, marketing, and usage of medical devices. While this legislation has allowed for vigorous growth in the medical device sector it has also resulted in insufficient oversight when approving many devices. Loopholes have been exploited by industry to bring more devices to the market in the pathway of least resistance in the swiftest and cheapest fashion. Modifications to current legislation should be sought to modernize medical device pathways to market and ensure patient safety.
